# Esophageal Cancer After Bariatric Surgery: Increasing Prevalence and Treatment Strategies

**DOI:** 10.1007/s11695-021-05679-1

**Published:** 2021-09-07

**Authors:** Victor D. Plat, Anne Kasteleijn, Jan Willem M. Greve, Misha D. P. Luyer, Suzanne S. Gisbertz, Ahmet Demirkiran, Freek Daams

**Affiliations:** 1 Department of Gastrointestinal Surgery, Amsterdam UMC, VU University Medical Center, De Boelelaan 1117, 1081 HV Amsterdam, Postbus 7075, 1007 MB Amsterdam, The Netherlands; 2 Department of Gastrointestinal Surgery, Zuyderland Medical Center, Heerlen, The Netherlands; 3 School of Nutrition and Translational Research in Metabolism (NUTRIM), Maastricht University Medical Center Maastricht, Maastricht, The Netherlands; 4 Department of Gastrointestinal Surgery, Catharina Hospital, Eindhoven, The Netherlands; 5 Department of Gastrointestinal Surgery, Rode Kruis Hospital, Beverwijk, The Netherlands

**Keywords:** Esophageal cancer, Bariatric surgery, Esophagectomy, Treatment

## Abstract

**Purpose:**

The number of bariatric procedures has increased exponentially over the last 20 years. On the background of ever-increasing incidence of esophageal malignancies, the altered anatomy after bariatric surgery poses challenges in treatment of these cancers. In this study, an epidemiological estimate is presented for the future magnitude of this problem and treatment options are described in a retrospective multicenter cohort.

**Methods:**

The number of bariatric procedures, esophageal cancer incidence, and mortality rates of the general population were used for epidemiological estimates. A retrospective multicenter cohort was composed; patients were treated in three large oncological centers with a high upper gastrointestinal cancer caseload. Consecutive patients with preceding bariatric surgery who developed esophageal cancer between 2014 and 2019 were included.

**Results:**

Approximately 3200 out of 6.4 million post bariatric surgery patients are estimated to have developed esophageal cancer between 1998 and 2018 worldwide. In a multicenter cohort, 15 patients with esophageal cancer or Barrett’s esophagus and preceding bariatric surgery were identified. The majority of patients had a history of Roux-en-Y gastric bypass (46.7%) and had an adenocarcinoma of the distal esophagus (60%). Seven patients received curative surgical treatment, five of whom are still alive at last follow-up (median follow-up 2 years, no loss to follow-up).

**Conclusion:**

Based on worldwide data, esophageal cancer development following bariatric surgery has increased over the past decades. Treatment of patients with esophageal cancer after bariatric surgery is challenging and requires a highly individualized approach in which optimal treatment and anatomical limitations are carefully balanced.

**Graphical abstract:**

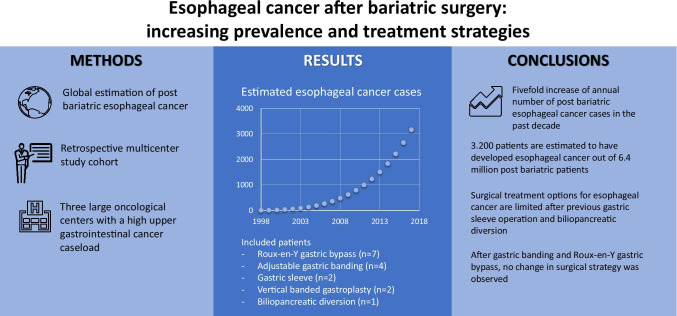

## Introduction

The worldwide prevalence of obesity has doubled between 1980 and 2015, resulting in numerous non-surgical (e.g., diet therapy, pharmaceutical therapy, lifestyle modification) and surgical approaches to manage obesity and the comorbidities it entails [1]. Bariatric surgery is considered the most effective treatment option in people suffering from morbid obesity [2]. Since the first laparoscopic gastric bypass was performed by Alan Wittgrove in 1993, the number of bariatric procedures performed per year has grown exponentially from 40,000 procedures performed in 1998 to an estimated 685,874 bariatric procedures performed in 2016 [3–5].

With an increasing incidence of morbid obesity, which is a known risk factor for esophageal adenocarcinoma (EAC), the incidence of EAC has increased dramatically in the Western countries over the past three decades [6]. The EAC incidence is expected to keep rising across high-income countries with the prediction that one in 100 men in the Netherlands and UK will be diagnosed with EAC before the age of 75 years by 2030 [7, 8]. Subsequently, bariatric surgery results in a decrease in incidence of obesity-related malignancies [9–11]. Recent evidence does not report an increased incidence of esophageal cancer after gastric sleeve or duodenal switch compared to non-surgical controls with obesity [12].

The altered anatomy due to preceding bariatric surgery poses significant treatment challenges. As regular surgical treatment of locally advanced esophageal tumors consists of esophagectomy with restoration of continuity by creation of a gastric conduit, this approach may be adjusted after a Roux-en-Y gastric bypass let alone impossible after creation of a gastric sleeve. Up to 2019, 39 esophageal malignancies after bariatric surgery from small single-center case series have been reported worldwide [13, 14]. The largest cohort of patients involves 8 patients, illustrating the low incidence in the individual practice [15, 16]. Hence, the lack of large multicenter studies or centralized registries likely underestimates the global incidence. As a substantial delay between bariatric surgery and the development of esophageal cancer is expected, the biggest peak incidence is probably yet to come.

The aim of this study was to estimate the global incidence of esophageal cancer following bariatric surgery and to create a multicenter study cohort to evaluate possible curative treatment options in this challenging surgical population.

## Methods

### Global Esophageal Cancer Estimates After Bariatric Surgery

Estimations of the annual numbers of bariatric procedures performed worldwide were available for 1998, 2003, 2008, 2011, 2013, 2014, and 2016 [4, 5, 17–21]. The missing years were interpolated and extrapolated, assuming a linear rise in the number of procedures compared to the previous and subsequent years. Because the esophageal cancer risk differs greatly for sex and age, different age categories were defined, separately for men and women using the reported age distribution by the International Federation for the Surgery of Obesity and Metabolic Disorders (IFSO) [22]: < 21, 21–30, 31–40, 41–50, 51–60, 61–70, > 70.

Obesity is known to increase esophageal cancer incidence [23]; however, bariatric surgery reduces the risk of obesity-related malignancies [24]. The incidence of esophageal cancer after bariatric surgery had not been confirmed to date. Current literature is limited to a single study, reporting no difference in incidence after bariatric surgery compared to non-surgical obese patients [12]. Therefore, the general esophageal cancer incidence, stratified by age groups and gender was used for the estimation. GLOBOCAN estimated crude rates of new esophageal cancer cases in 2018 were used [25]. General mortality rates in United Nations more developed regions were extracted from the *World Population Prospects* and separately calculated for the different age categories, men and women [26].

For each separate year (1998 until 2018), the “population at risk” was estimated, which was defined as subjects following bariatric surgery subtracted by the estimated esophageal cancer cases and estimated diseased subjects. The population at risk was divided according to gender and previously mentioned age categories, which were multiplied by the corresponding esophageal cancer crude rate and mortality rates. This was repeated until the estimated esophageal cancer cases of the period 1998 until 2018 were calculated for each particular year.

### Multicenter Study Cohort

The study describes a retrospective multicenter study cohort in which patients treated in the Amsterdam UMC (locations VUmc and AMC) and the Catharina Hospital Eindhoven in the Netherlands were included, having a combined annual volume of approximately 200 esophagectomies. Patients with any stage esophageal cancer, diagnosed between 2014 and 2019, preceded by bariatric surgery (i.e., gastric sleeve, Roux-en-Y gastric bypass, one anastomosis gastric bypass, (adjustable) gastric banding, vertical banded gastroplasty, or biliopancreatic diversion with or without duodenal switch) were included. All patients had symptoms of esophageal cancer and underwent a preoperative staging gastroscopy and PET-CT imagining. Patient, cancer, and surgical characteristics were extracted from electronic records.

### Statistical Analysis and Ethical Considerations

Continuous variables were expressed as median with Interquartile rang (IQR) and frequency percentages were calculated for dichotomous variables. IBM SPSS statistics (version 26 IBM Corp. Armonk, NY) was used for standard statistical analysis. This study was conducted in accordance with the Declaration of Helsinki [27] and reported according to the STROBE (Strengthening the Reporting of Observational Studies in Epidemiology) statement [28]. The medical ethics committee of the Amsterdam UMC and Catharina Hospital approved this study. All living subjects have been provided the opportunity to opt-out and received a written no objection letter.

## Results

### Global Esophageal Cancer Estimates After Bariatric Surgery

**Fig. 1:**
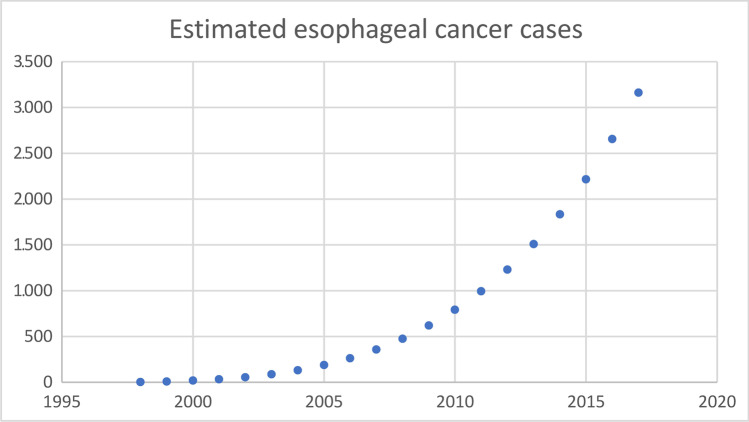
Estimated esophageal cancer cases after bariatric surgery between 1998 and 2018

The global number of bariatric procedures has been extracted from the available estimates [4, 5, 17–21]. The remaining years were calculated, leading to an estimated 6.4 million procedures performed worldwide between 1998 and 2018. Global patients characteristics were derived from recent IFSO reports [22], which showed that 74% of patients were female and 26% were males. The median age was 42 (IQR 33–50) for females and 44 (IQR 34–52) for male patients. These characteristics were applied to the total population at risk and multiplied by corresponding esophageal cancer crude rates. Table 1 shows the estimated annual bariatric procedures, population at risk, annual esophageal cancer cases, and total esophageal cancer cases for each separate year between 1998 and 2018. The total estimated esophageal cancer cases are visualized in Fig. 1.

**Table 1:** For each separate year between 1998 and 2018 the annual bariatric procedures, population at risk, annual esophageal cancer cases and accumulated esophageal cancer were calculated

Year	Estimated annual bariatric procedures	Estimated population at risk	Estimated annual esophageal cancer cases	Estimated total esophageal cancer cases
**1998**	**40,000**	40,000	2.2	2
1999	61,260	101,147	5.5	8
2000	82,520	183,381	9.9	18
2001	103,781	286,641	15.5	33
2002	125,041	410,868	22.3	55
**2003**	**146,301**	556,002	31.3	87
2004	185,885	740,261	43.1	130
2005	225,469	963,510	57.5	187
2006	265,053	1,225,615	74.8	262
2007	304,637	1,526,439	94.7	357
**2008**	**344,221**	1,865,847	118.7	476
2009	343,070	2,202,904	144.6	620
2010	341,919	2,537,536	172.2	792
**2011**	**340,768**	2,869,667	201.5	994
2012	404,689	3,264,295	236.2	1.230
**2013**	**468,609**	3,721,161	277.5	1.508
**2014**	**579,517**	4,286,902	326.9	1.834
2015	632,696	4,903,384	381.3	2.216
**2016**	**685,874**	5,570,367	440.6	2.656
2017	721,756	6,270,320	505.7	3.162

The available annual numbers of bariatric procedures performed worldwide are highlighted in bold

### Patient Selection and Characteristics

All patients who were discussed in a multidisciplinary meeting for treatment of esophageal cancer between 2014 and 2019 in the Amsterdam UMC (location AMC and VUmc) and Catharina Hospital Eindhoven (*n* = 3.833) were analyzed for a history with bariatric surgery. Fifteen patients with esophageal cancer and preceding bariatric surgery were identified, of which eight (53.3%) were male. Preceding bariatric surgery consisted of Roux-en-Y gastric bypass (seven patients, 46.7%), gastric banding (four patients, 26.7%), gastric sleeve (two patients, 13.3%), vertical banded gastroplasty (one patient, 6.7%), and biliopancreatic diversion (one patient, 6.7%). Median age at bariatric surgery was 51.5 years (IQR 36.0–57.3). Median BMI preceding bariatric surgery was 47.6 (IQR: 40.6–56.8).

**Fig. 2:**
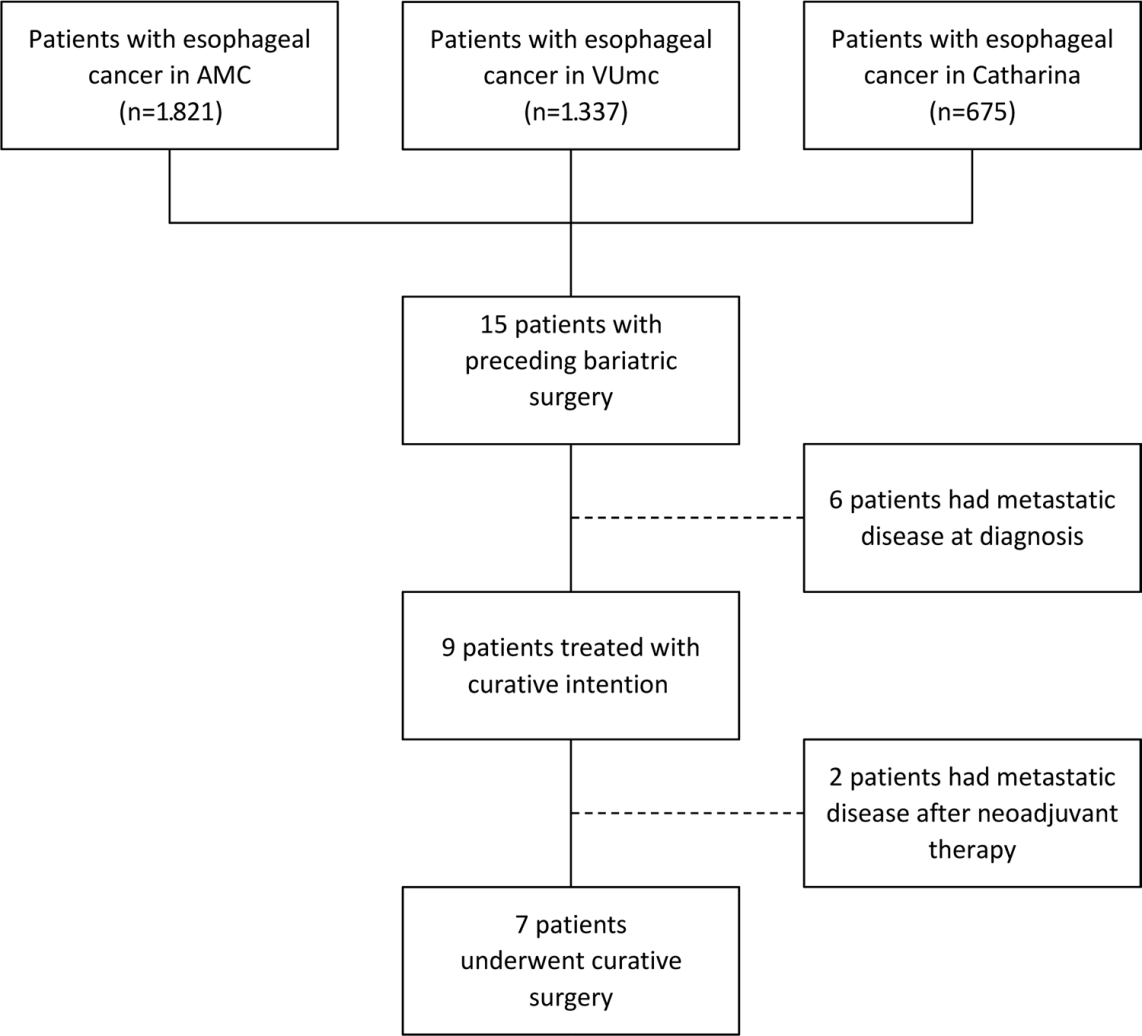
Flow-chart of the identification of patients

The majority of patients had an EAC of the distal esophagus (nine patients, 60%), other types and locations consisted of mid EAC (one patient, 6.7%), adenocarcinoma of the gastro-esophageal junction (one patient, 6.7%), squamous cell carcinoma of the gastro-esophageal junction (one patient, 6.7%), squamous cell carcinoma of the proximal esophagus (one patient, 6.7%), and Barrett’s esophagus (two patients, 13.3%). Median age at esophageal cancer diagnosis was 58.0 years (IQR 50.0–64.0). Six patients were diagnosed with metastatic disease. Nine patients started treatment with a curative intention, two of whom developed metastatic disease after neoadjuvant chemoradiotherapy. Seven remaining patients received curative treatment, five of whom are still alive at last follow-up (median follow-up 2 years, IQR 1–3 years). All patient characteristics are summarized in Table 2 and no patients were lost to follow-up. A flow-chart of the identification of patients is depicted in Fig. 2.

**Table 2:** Patient characteristics. BMI indicates body mass index; EAC, esophageal adenocarcinoma; GEJ, gastro-esophageal junction; SCC, squamous cell carcinoma

Patient	Gender	Bariatric surgery	Age bariatric surgery	Age cancer diagnosis	Age diff	Cancer type and location	TNM	BMI bariatric surgery	BMI cancer diagnosis	Surgery
001	Male	Roux-en-Y gastric bypass	52	54	2	EAC GEJ	T3N3Mx	43.1	26.2	No
002	Female	Roux-en-Y gastric bypass	54	61	7	Distal EAC	T3NxMx	-	23.4	No
003	Male	Roux-en-Y gastric bypass	63	70	7	Distal EAC	T3N2M1	62.3	23.9	No
004	Female	Roux-en-Y gastric bypass	47	-	-	Barrett’s	-	33.0	20.5	No
005	Female	Roux-en-Y gastric bypass	58	64	6	Distal EAC	T3N1M0	69.9	23.5	Yes
006	Female	Roux-en-Y gastric bypass	54	63	9	SCC GEJ	T3N3Mx	-	31.3	No
007	Female	Roux-en-Y gastric bypass	27	41	14	Proximal SCC	T3N1M0	-	30.5	Yes
008	Male	Adjustable gastric banding	33	47	14	Distal EAC	T1N0Mx	54.9	30.8	Yes
009	Male	Adjustable gastric banding	37	47	10	Distal EAC	T3N1M1	41.4	30.0	No
010	Female	Adjustable gastric banding	58	68	10	Distal EAC	T3N0M0	51.3	35.0	Yes
011	Male	Adjustable gastric banding	43	57	14	Distal EAC	TxN1M1	-	-	No
012	Male	Gastric sleeve	57	59	2	Distal EAC	T4N3M1	38.1	23.7	No
013	Male	Gastric sleeve	51	-	-	Barrett’s	C0M1*	-	39.1	Yes
014	Male	Vertical banded gastroplasty	25	53	28	Distal EAC	T1N1M0	48.8	28.4	Yes
015	Female	Biliopancreatic diversion	-	64	-	Mid EAC	T3N0M0	46.3	25.5	Yes

### Technical Considerations

Seven patients underwent surgery with curative intent, surgical specifics, and outcomes are summarized in Table 3.

**Table 3:** Surgical specifics, short- and long-term outcome of patient who underwent surgery with curative intent. CRT indicates chemoradiotherapy

Patient	Bariatric surgery	Treatment	Outcomes	Radicality	Follow-up
005	Roux-en-Y gastric bypass	Neoadjuvant CRT and minimally invasive Ivor-Lewis esophagectomy	Complications after both chemoradiotherapy and surgery	R1	Metastasis 1 year after diagnosis
007	Roux-en-Y gastric bypass	Neoadjuvant CRT and minimally invasive McKeown esophagectomy	Proximal necrosis gastric conduit requiring takedown of anastomosis, empyema, Clagett thoracotomy, ICU acquired weakness	R0	Disease-free 4 years after diagnosis
008	Adjustable gastric banding	Endoscopic mucosal resection and radiofrequency ablation	No complications	R0	Disease-free 2 years FU
010	Adjustable gastric banding	Neoadjuvant CRT and minimally invasive Ivor-Lewis esophagectomy	No complications	R0	Disease-free 3 years after diagnosis
013	Gastric sleeve	Endoscopic mucosal resection and radiofrequency ablation	No complications	R0	Disease-free 2 years after diagnosis
014	Vertical banded gastroplasty	Neoadjuvant CRT and hybrid Ivor-Lewis esophagectomy	Chyle leakage, atrial fibrillation, pneumonia	R0	Metastasis 1 year after diagnosis
015	Biliopancreatic diversion	Neoadjuvant CRT and open esophagectomy with colon interposition	Anastomotic leakage requiring surgery, respiratory insufficiency after pneumonia	R0	Disease-free 2 years after diagnosis

Roux-en-Y gastric bypass *(two patients):* Two patients presented with a T3N1M0 distal esophageal tumor with preceding Roux-en-Y gastric bypass for which neoadjuvant chemoradiotherapy, followed by a resection was advised. After intraoperative evaluation of adequate blood supply of the gastric remnant a gastric conduit was formed, with adequate length for cervical and intrathoracic anastomosis in both patients. There were multiple postoperative complications in both patients, among which was proximal necrosis of the gastric conduit in one patient. This patient is alive at follow-up 4 years after diagnosis, whereas the other patient showed metastatic disease 1 year after diagnosis.

Adjustable gastric banding *(two patients):* One patient had a medical history of placement and removal of an adjustable gastric banding and developed a T3N0M0 EAC a few years later. Curative neoadjuvant radiotherapy and esophagectomy with gastric conduit formation was feasible. Intraoperatively, the blood supply and length of the gastric conduit were not compromised. Postoperative course was uncomplicated. The other patient was diagnosed with a T1N0M0 EAC and was treated with endoscopic mucosal resection (EMR) and radiofrequency ablation (RFA) without postoperative complications. Both patients had no sign of recurrence at the last follow-up.

Gastric sleeve *(one patient)*: This patient was diagnosed with a Barrett’s esophagus with Prague classification C0M1 and received treatment with EMR and RFA without postoperative complications and without recurrent disease at the last follow-up 2 years later.

Vertical banded gastroplasty *(one patient):* This patient with a T1N1M0 EAC after vertical banded gastroplasty was treated with neoadjuvant chemoradiotherapy and two-stage esophagectomy with gastric conduit formation using open approach for the abdominal phase and minimally invasive thoracoscopic approach. Both blood supply and length of the conduit were not compromised. Multiple postoperative complications (atrial fibrillation, chylus leakage, and pneumonia) followed and this patient developed metastatic disease 1 year later.

Biliopancreatic diversion *(one patient):* One patient with preceding vertical banded gastroplasty and previous conversion to biliopancreatic diversion developed a T3N0M0 EAC. Curative intent with neoadjuvant chemoradiotherapy followed by an esophagectomy was executed. Intraoperatively, the gastric remnant was not suitable for formation of a gastric conduit; therefore, a colonic interposition was performed. Postoperative recovery was complicated by anastomotic leakage requiring surgery. This patient was disease free at the last follow-up 2 years later.

## Discussion

This study estimated an increased number of annual esophageal cancer cases in patients with a history of bariatric surgery over the past decades. Subsequently, 15 patients were identified of which seven were treated with curative intent. The altered gastrointestinal anatomy of these patients challenged the treating physicians to determine alternative treatment strategies. This study reported clinical preoperative considerations and cancer stage as main determining factors, and showed that patients were treated both with surgical and non-surgical intervention using a highly individualized approach.

Obesity is a well-known risk factor for esophageal cancer [6], while bariatric surgery, on the other hand, is associated with a reduced incidence in overall cancer incidence and obesity-related cancer incidence [9–11, 29–31]. Next to this, weight loss is also known for reducing chronic reflux and might therefore also have a long-term preventive effect on the occurrence of esophageal cancer. The incidence of esophageal cancer after bariatric surgery has not been determined in large longitudinal cohort studies and current evidence is limited, reporting no difference in incidence after bariatric surgery compared to non-surgical obese patients [12]. In present study cohort, no conclusions regarding this incidence could be drawn due to the lack of a denominator, as two out of three participating centers have a high upper gastrointestinal cancer caseload but do not perform bariatric surgery. Next to this, it is conceivable that patients that were operated in the third center that performs both types of surgery have presented with an esophageal malignancy in a hospital beyond our scope. Furthermore, the lack of an adequate sample size would have hampered an accurate calculation. As for the annual number of esophageal cancer cases in patients with a history of bariatric surgery, the global estimates show a fivefold increase between 2007 and 2017 (approximately 100 to 500 cases). This indicates that not the incidence of esophageal cancer after bariatric surgery per se, but the fast increasing number of bariatric procedures performed will determine the annual number of esophageal cancer cases in patients with a history of bariatric surgery in the future.

Curative intent surgical approach with gastric conduit replacement of the esophagus remained possible in patients with preceding Roux-en-Y gastric bypass, adjustable gastric banding, and vertical banded gastroplasty. This was possible after assertion of good blood supply and adequate length of the gastric conduit. Since the gastric remnant is usually preserved at the greater curvature during these procedures and both the right gastric artery and the right gastroepiploic artery are routinely untouched [32]. Colonic interposition or high esophagojejunostomy offer an alternative for restoring continuity and the patient should be counseled and prepared for this possibility [33]. The presented cohort shows a great variety in type of bariatric surgery, disease type (EAC, SCC, and Barret’s esophagus), cancer location, cancer stage, and time interval between bariatric surgery and cancer diagnosis. This heterogeneity created a cohort which is a good reflection of current practice, however resulted in the inability to perform statistical tests and address the impact of different treatment options.

Given their less invasive nature, especially compared to esophagectomy with colonic interposition for restoration of continuity, organ-sparing therapies such as definitive chemoradiotherapy and EMR should be considered as alternative treatment [34, 35]. Especially in patients with poor condition, when damage to the stomach or blood supply is expected or confirmed or in patients with high squamous esophageal tumors that require a long conduit, this may be the preferred treatment strategy.

Approximately half of the available evidence has been published within the past 2 years, illustrating a recent growth in interest [12, 36–40]. The literature mainly involves small case series, none of which included more than eight patients, which describe similar treatment options [15, 16, 33, 41–43]. The majority of curative treatment options comprise esophagectomy followed by gastric conduit formation after Roux-en-Y gastric bypass or, for junction tumors total gastrectomy with distal esophagectomy followed by esophagojejunostomy, a treatment option that was not performed in any of the patients in current cohort [37]. Definitive chemoradiotherapy is a non-surgical option that has been used after gastric sleeve surgery, which is a recognized alternative however not used in this cohort [35, 44].

The complexities and individual approach require a highly specialized approach, in which gastroenterologists and surgeons should not only be experts in oncologic upper gastrointestinal treatment but also require excellent knowledge and skill to understand and overcome the anatomic challenges. Therefore, centralization of care into an oncobariatric center might lead to improved oncological and surgical outcomes.

This study was subjected to limitations. First, the number of bariatric procedures was estimated based international surveys, having a higher reported response rate in 2016 compared to earlier years. This might have led to an underestimation of bariatric procedures. Second, data was not available for each separate year and missing years were interpolated and extrapolated. Third, esophageal cancer incidence was estimated using incidences of the general population, a potential effect of obesity, bariatric surgery, or weight loss on esophageal cancer development has not been considered. Fourth, since the number of healthy post bariatric patients in the Netherlands is not known, and the included number of patients in this study consist of a sample of three oncological centers, the true incidence of esophageal cancer after bariatric surgery could not be calculated. This inability also holds for the incidence of esophageal cancer after bariatric surgery compared to obese patients that were not operated on. Fifth, not all preoperative considerations and intraoperative technical specifics could be extracted due to the retrospective design of the study. Finally, although this was a multicenter study involving large oncological centers with a high upper gastrointestinal cancer caseload, a limited number of patients were identified. Hence, a large international retrospective study, Oesophago-Gastric Malignancies after Obesity Surgery (OGMOS), is currently in progress to further elucidate the magnitude of this problem. However, a large prospective international auditing initiative, ideally linked to IFSO data, is needed to estimate the true clinical incidence of esophageal cancer after bariatric surgery.

In conclusion, post bariatric esophageal cancer has increased over the past decades and is projected to continue to increase in the future. This study reported clinical preoperative considerations concerning the altered gastrointestinal anatomy that requires a careful and individual, both surgical and non-surgical approach as reconstruction options may be limited.

## References

[1] Chooi YC, Ding C, Magkos F (2019). The epidemiology of obesity. Metabolism.

[2] Ozsoy Z, Demir E (2018). Which bariatric procedure is the most popular in the world? A Bibliometric Comparison. Obes Surg.

[3] Faria GR (2017). A brief history of bariatric surgery. Porto Biomed J.

[4] Angrisani L, Santonicola A, Iovino P (2018). IFSO worldwide survey 2016: primary, endoluminal, and revisional procedures. Obes Surg.

[5] Scopinaro N (1998). The IFSO and obesity surgery throughout the world. Obes Surg.

[6] Hoyo C, Cook MB, Kamangar F (2012). Body mass index in relation to oesophageal and oesophagogastric junction adenocarcinomas: a pooled analysis from the International BEACON Consortium. Int J Epidemiol.

[7] Arnold M, Laversanne M, Brown LM (2017). Predicting the future burden of esophageal cancer by histological subtype: international trends in incidence up to 2030. Am J Gastroenterol.

[8] Coleman HG, Xie SH, Lagergren J (2018). The epidemiology of esophageal adenocarcinoma. Gastroenterology.

[9] Ostlund MP, Lu Y, Lagergren J (2010). Risk of obesity-related cancer after obesity surgery in a population-based cohort study. Ann Surg.

[10] Schauer DP, Feigelson HS, Koebnick C (2019). Bariatric surgery and the risk of cancer in a large multisite cohort. Ann Surg.

[11] Wiggins T, Antonowicz SS, Markar SR (2019). Cancer risk following bariatric surgery-systematic review and meta-analysis of national population-based cohort studies. Obes Surg.

[12] Andalib A, Bouchard P, Demyttenaere S, Ferri LE, Court O (2021). Esophageal cancer after sleeve gastrectomy: a population-based comparative cohort study. Surg Obes Relat Dis..

[13] Musella M, Berardi G, Bocchetti A (2019). Esophagogastric neoplasms following bariatric surgery: an updated systematic review. Obes Surg.

[14] Scozzari G, Trapani R, Toppino M (2013). Esophagogastric cancer after bariatric surgery: systematic review of the literature. Surg Obes Relat Dis.

[15] Burton PR, Ooi GJ, Laurie C (2016). Diagnosis and management of oesophageal cancer in bariatric surgical patients. J Gastrointest Surg.

[16] Maret-Ouda J, Tao W, Mattsson F (2017). Esophageal adenocarcinoma after obesity surgery in a population-based cohort study. Surg Obes Relat Dis.

[17] Buchwald H, Williams SE (2004). Bariatric surgery worldwide 2003. Obes Surg.

[18] Buchwald H, Oien DM (2009). Metabolic/bariatric surgery Worldwide 2008. Obes Surg.

[19] Buchwald H, Oien DM (2013). Metabolic/bariatric surgery worldwide 2011. Obes Surg.

[20] Angrisani L, Santonicola A, Iovino P (2015). Bariatric Surgery worldwide 2013. Obes Surg.

[21] Angrisani L, Santonicola A, Iovino P (2017). Bariatric surgery and endoluminal procedures: IFSO worldwide survey 2014. Obes Surg.

[22] Himpens J, Ramos A, Welbourn R, et al. 4th IFSO Global Registry Report. Available from: https://www.ifso.com/pdf/4th-ifso-global-registry-report-last-2018.pdf. 2018. Accessed 01-03-2021.

[23] Alsop BR, Sharma P (2016). Esophageal Cancer. Gastroenterol Clinics North Am.

[24] Ebrahimi R, Kermansaravi M, Khalaj A (2019). Gastro-intestinal tract cancers following bariatric surgery: a narrative review. Obes Surg.

[25] World Health Organization. Cancer Today. Available from: https://gco.iarc.fr/today/home. 2018. Accessed 01-03-2021.

[26] Nations U. World population prospects 2019. Available from: https://population.un.org/wpp/DataQuery/. Accessed 01-03-2021.

[27] World Medical Association Declaration of Helsinki (2013). ethical principles for medical research involving human subjects. JAMA.

[28] von Elm E, Altman DG, Egger M (2007). Strengthening the Reporting of Observational Studies in Epidemiology (STROBE) statement: guidelines for reporting observational studies. BMJ (Clinical research ed).

[29] Adams TD, Stroup AM, Gress RE (2009). Cancer incidence and mortality after gastric bypass surgery. Obesity (Silver Spring, Md).

[30] Christou NV, Lieberman M, Sampalis F (2008). Bariatric surgery reduces cancer risk in morbidly obese patients. Surg Obes Relat.

[31] Schauer DP, Feigelson HS, Koebnick C (2017). Association between weight loss and the risk of cancer after bariatric surgery. Obesity (Silver Spring, Md).

[32] Wittgrove AC, Clark GW, Tremblay LJ (1994). Laparoscopic gastric bypass, Roux-en-Y: preliminary report of five cases. Obes Surg.

[33] Rossidis G, Browning R, Hochwald SN (2014). Minimally invasive esophagectomy is safe in patients with previous gastric bypass. Surg Obes Relat Dis.

[34] Shah PM, Gerdes H (2015). Endoscopic options for early stage esophageal cancer. J Gastrointest Oncol.

[35] Voeten DM, den Bakker CM, Heineman DJ (2019). Definitive chemoradiotherapy versus trimodality therapy for resectable oesophageal carcinoma: meta-analyses and systematic review of literature. World J Surg.

[36] Latzko M, Ahmed B, Awad Z (2021). Minimally invasive Ivor-Lewis esophagectomy for esophageal cancer after gastric bypass. Ann Surg Oncol.

[37] Genco A, Castagneto-Gissey L, Lorenzo M (2021). Esophageal adenocarcinoma after sleeve gastrectomy: actual or potential threat? Italian series and literature review. Surg Obes Relat Dis.

[38] Alaber O, Mansoor E, Perez LKM (2021). High grade dysplasia or esophageal adenocarcinoma in patients with a history of Roux-en-Y gastric bypass surgery: a case series. Endoscopy.

[39] Bevilacqua LA, Obeid NR, Yang J (2020). Incidence of GERD, esophagitis, Barrett’s esophagus, and esophageal adenocarcinoma after bariatric surgery. Surg Obes Relat Dis.

[40] Janse P, Nafteux P, Lannoo M (2019). Esophageal squamous cell carcinoma after adjustable gastric banding. Obe Surg.

[41] Allen JW, Leeman MF, Richardson JD (2004). Esophageal carcinoma following bariatric procedures. JSLS.

[42] Kulaylat AN, Sahajwani S, Staveley-O'Carroll KF (2013). Reconstructive options for gastroesophageal junction adenocarcinoma after Roux-en-Y gastric bypass. J Thoracic Cardiovasc Surg.

[43] Melstrom LG, Bentrem DJ, Salvino MJ (2008). Adenocarcinoma of the gastroesophageal junction after bariatric surgery. Am J Surg.

[44] Scheepers AF, Schoon EJ, Nienhuijs SW (2011). Esophageal carcinoma after sleeve gastrectomy. Surg Obes Relat Dis.

